# Bilateral sequential theta burst stimulation in depressed veterans with service related posttraumatic stress disorder: a feasibility study

**DOI:** 10.1186/s12888-022-03729-1

**Published:** 2022-02-03

**Authors:** Thelepa Vaithianathan, Mervin Blair, Vanessa Soares, Yuri E. Rybak, Lena Palaniyappan, J Don Richardson, Amer M. Burhan

**Affiliations:** 1 Ontario Shores Centre for Mental Health Sciences, 700 Gordon Street, Whitby, Ontario L1N 9X4 Canada; 2 Lawson Health Research Institute, London, Ontario Canada; 3 MacDonald Franklin OSI Research Centre, London, Ontario Canada; 4 Department of Psychiatry, Schulich School of Medicine and Dentistry, University of Western Ontario, Ontario London, Canada; 5 Robarts Research Institute, University of Western Ontario, London, Ontario Canada; 6 Department of Psychiatry, Temerty Faculty of Medicine, University of Toronto, Toronto, Ontario Canada

**Keywords:** TRD, PTSD, theta burst stimulation, Military, Veterans

## Abstract

**Background:**

Depression comorbid with posttraumatic stress disorder (PTSD) can be disabling and treatment resistant. Preliminary evidence suggests that repetitive transcranial magnetic stimulation (rTMS), may have a role in helping these patients. There are only few published studies using different rTMS paradigms including bilateral intermittent theta burst (iTBS) and low frequency rTMS.

**Methods:**

In this small cohort observation study, we examined the efficacy of bilateral sequential theta-burst stimulation (bsTBS) in 8 treatment resistant depression (TRD) military veterans with PTSD comorbidity stemming from military service experience.

**Results:**

bsTBS was generally well tolerated and resulted in 25% and 38% remission and response rates on Depression scores respectively; 25% remission and response rate on PTSD scores.

**Discussion:**

This study demonstrates preliminary feasibility and safety of bsTBS in TRD with comorbid military service related PTSD.

We concluded that this paradigm might hold promise as a therapeutic tool to help patients with TRD co-morbid with military service related PTSD. Further adequately powered studies to compare rTMS treatment paradigms in this patient group are warranted.

## Introduction

Posttraumatic stress disorder (PTSD) can be comorbid with other illnesses and can cause significant impairment [1]. Current interventions for PTSD are limited in efficacy [2]. Consequently, there is a need for new interventions.

Repetitive transcranial magnetic stimulation (rTMS) is a relatively non-invasive procedure increasingly used to treat psychiatric disorders. A meta-analysis of 17 studies demonstrated that right dorsolateral prefrontal cortex (DLPFC) high frequency stimulation is the most effective treatment for PTSD [3]. Standard high frequency rTMS requires 37.5 min to deliver. Intermittent theta burst stimulation (iTBS) can be delivered in 3 min and is non-inferior to high frequency rTMS in treating treatment resistant depression (TRD) [4].

Two studies examined iTBS in PTSD and showed a reduction in depression and PTSD symptoms [5, 6]. The site of stimulation differed between the two studies (right iTBS and bilateral iTBS respectively). A recent study by Philip et al. [7] showed that rTMS, at 5-Hz, was superior to iTBS in the treatment of PTSD symptoms when both are applied to the left DLPFC only. To treat PTSD, a meta-analysis by Kan et al. [8] supported right-sided inhibitory protocols, although at somewhat lower effect-size than left-sided DLPFC stimulation.

For depression, especially TRD, several lines of evidence support the use of left DLPFC stimulation [9, 10]. Evidence suggests that patients with depressive disorders present with frontal activity asymmetry (right > left) [11, 12]. While this still needs to be clarified further given the heterogeneity of studies, rTMS studies utilizing right inhibitory stimulation followed by left excitatory stimulation have shown to be effective in TRD [9, 10, 13, 14]. Thus, in the present retrospective study in which bilateral stimulation was applied, we reasoned that inhibitory protocol (cTBS) on the right DLPFC would alleviate PTSD symptoms, while left-sided excitatory protocol (iTBS) would help TRD symptoms. With respect to burst sequence, Plewnia et al. [15] found that the bilateral TBS protocol, that we used, was safe and superior to sham stimulation for the treatment of major depression. Further, we recently reported good efficacy and tolerability for bilateral sequential theta-burst applications in TRD [16].

 In the current retrospective chart review, we examined the efficacy and safety of bilateral sequential TBS (bsTBS) in patients with TRD and co-morbid PTSD. We investigated the notion that right sided cTBS would improve PTSD symptoms and left sided iTBS would reduce depressive symptoms [17, 18].

## Methods

 This is a retrospective chart review and was conducted in accordance with the Declaration of Helsinki and was approved by the Research Ethics Board at the University of Western Ontario (project ID: 116,274). Data was collected retrospectively on 15 TMS clinic patients referred for TRD and PTSD, as such, informed consent was not required for data collection, however was obtained prior to treatment. The data sets used during the current study are available from the corresponding author upon request.

### Inclusion

18 years and older, diagnosed with TRD and PTSD and received a minimum of 10 bsTBS sessions.

The patients were referred from the Operational Stress Injury Clinic, having the diagnosis of military related PTSD. The diagnosis of PTSD was made by a psychiatrist through a comprehensive clinical assessment in combination with the Clinical Administered PTSD Scale (CAPS) and the self-administered PTSD checklist (PCL-5). The diagnosis of TRD was obtained by using clinical impression of a major depressive episode with partial or no response to at least two optimum courses of antidepressant trials per referring psychiatrist and as confirmed on intake by the Therapeutic Brain Stimulation Clinic.

### Exclusion

 3 received different rTMS paradigms and 4 patients withdrew (3 due to side effects including headaches and 1 due to scheduling conflicts).

Eight patients received 19 to 30 sessions over 4 – 6 weeks.

Baseline and post bsTBS assessments were obtained from the HAMD-17, Generalized Anxiety Disorder Scale (GAD-7), Patient Health Questionnaire (PHQ-9), PTSD Checklist for DSM-5 (PCL-5), and Clinical Global Impression Severity-Improvement Scale (CGI-S, CGI-I).

The primary outcome was the change in HAMD-17 score from baseline to the end of treatment. Secondary outcomes included change in PCL-5, PHQ-9, GAD-7 and CGI scores. Clinical response was defined as >= 50% reduction of baseline scores on the HAMD-17, PCL-5, PHQ-9 and GAD-7, whereas remission was defined by HAMD-17 score of <=7 [19]. We opted to define remission for the remaining scales when scores fell below the normative cut-offs: PCL-5 score of <31-33 [20], PHQ-9 score of <5 [21] and GAD-7 score of <5 [22].

The TMS Machine (Magstim**®** Rapid 2, UK) was used to apply the paradigm published previously [16]. Briefly, 600 pulses of continuous TBS (cTBS) was applied to F4 location and subsequently iTBS to F3 location with burst frequency between 40 and 50 Hz. We used Beam F3 method to identify F3 location on the left side [23] and the corresponding F4 location on the right side. Resting motor threshold (RMT) was determined by eliciting a visible muscle twitch in the thumb or index finger on the right hand in 3 of 5 stimulation trials. Energy between 80 and 120% of the RMT was used depending on tolerability.

## Results

Eight male patients between ages 30 and 60 were included. All 8 patients received combination (2+ antidepressants) or augmentation (antidepressant plus non-antidepressant) treatment during their lifetime and were receiving medication for depression at time of treatment. The demographics of each patient with number of sessions, stimulus parameters and change in severity of illness is presented in Table 1.

**Table 1:** Demographics and parameters of bilateral rTMS treatments received by participants

	Demographics	Parameters				Rating scales (Pre/Post)				Clinical Impression
ID	Age (sex)	%RMT	Left (F3)	Right (F4)	Sessions	HAMD-17	PHQ-9	GAD-7	PCL-5	CGI (S/C)
1	59(M)	80	iTBS	cTBS	20	16/17	22/21	18/18	-/-	-/3
2	53(M)	100	iTBS	cTBS	20	25/17	23/18	18/15	-/-	5/3
3	56(M)	100	iTBS	cTBS	30	18/12	23/13	18/9	42/40	4/2
4	39(M)	100	iTBS	cTBS	27	29/11	19/16	16/12	62/47	6/3
5	50(M)	100	iTBS	cTBS	19	23/3	13/2	8/0	45/6	6/1
6	56(M)	80	iTBS	cTBS	29	26/7	17/6	14/3	51/21	5/1
7	43(M)	100	iTBS	cTBS	30	21/20	17/17	14/10	50/41	5/4
8	34(M)	100	iTBS	cTBS	30	26/16	17/10	11/7	48/-	5/2

Note: Scores for pre-treatment and post-treatment indicate changes to psychiatric scale scores showing changes due to treatment. Severity of illness is scored using the Clinical Global Impression (CGI) scale. CGI-S indicates the score given by the physician, ranging from 1 (normal, not at all ill) to 7 (among the most extremely ill patients in the population). The CGI-C indicates the change in CGI score after the treatment. Scoring for this scale ranges from 1 (very much improved) to 7 (very much worse)

*iTBS* intermittent theta-burst stimulation delivered at 3 s trains of 3 × 50hz bursts at 5 hz burst frequency with 8 inter-train seconds delay, 67 trains=603 pulses total

*cTBS* Continuous train of 200 × 3 × 50 hz bursts at 5 hz burst frequency, with no intertrain delay=600 pulses total

Following bsTBS, 38% and 25% met response and remission criteria respectively based on HAMD-17 scores. Results were comparable based on self-rating for PCL-5 (25% response and remission), GAD-7 (38% and 25% respectively), and PHQ-9 (25% and 13% respectively).

**Fig. 1:**
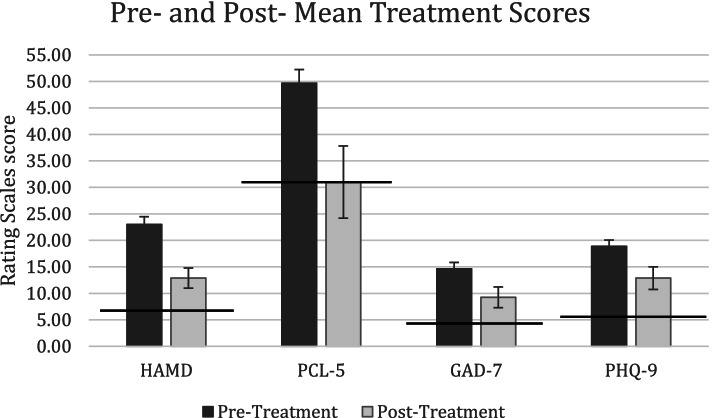
Mean pre- and post-treatment scores (standard error) following first course of bilateral sequential theta burst stimulation on Hamilton Depression Rating Scale (HAMD-17), PTSD Checklist for DSM-5 (PCL-5), Generalized Anxiety Disorder Scale (GAD-7), and Patient Health Questionnaire (PHQ-9). Solid horizontal lines represent clinical cut-offs for each scale

Pre- to post-treatment improvement was evident for HAMD-17 (44%; Cohen’s d = 1.325), PCL-5 (37.59%; Cohen’s d = 1.370), PHQ-9 (31.78%; Cohen’s d = 1.445) and GAD-7 (36.78%; Cohen’s d = 1.586) (Fig. 1).

The average CGI-S score at baseline was 5.14 (SD = 0.64), (range from markedly ill to severely ill). The average change in CGI-I score at post-treatment was 2.38 (SD = 0.99) (range from minimally improved to much improved).

Most patients experienced mild side effects (muscle contraction, pain/discomfort, scalp irritation and changes in systolic blood pressure). No patient experienced syncope or seizures.

Three patients returned for additional bsTBS after 4, 9 and 17 months, 2/3 of patients responded again to the same paradigm.

## Discussion

In this retrospective study, we examined the efficacy and safety of bsTBS in 8 patients referred to a TMS clinic for TRD comorbid with PTSD. The treatment was well tolerated and resulted in improvement in depressive, anxiety and PTSD symptoms. We chose this paradigm given the evidence of right-left activity asymmetry in depression. In a recent review Bruder et al. [12], outlined the evidence from multiple sources indicating relative hypoactivity of left DLPFC in depression especially in the presence of anxiety symptoms and in relation of the failure to inhibit the right amygdala in response to emotional stimuli. We speculated that using inhibitory TBS on right DLPFC followed by excitatory TBS on the left DLPFC would result in improvement of depressive and anxiety symptoms in our cohort.

Further support for the right DLPFC target in PTSD comes from the largest sham-controlled study of its kind including 50 PTSD patients, whereby Philip et al. [5] applied iTBS to right DLPFC targeting PTSD symptoms, and found a small effect size on PCL-5 after two weeks of treatment (Cohen’s d = 0.34).

Two recent papers published sub-sequent to our study, supported that right DLPFC is a good target to treat PTSD symptoms regardless if it is excitatory or inhibitory (McGirr [24], Khan [8]). There is evidence to suggest that bilateral rTMS might be superior to unilateral high frequency rTMS in remission rate in TRD [13, 14]. There is paucity of studies that used bilateral TBS to treat depression or PTSD.

On the other hand, a small observation study similar to ours, including 8 PTSD patients (7 males) [6], utilized bilateral iTBS and observed a treatment effect on both PTSD (clinician-administered PTSD Scale for DSM5, CAPS-5, clinician rated structured interview, Cohen’s d = 1.78) and depressive symptoms (HAMD-17 Cohen’s d = 1.16). Our sample showed a slightly higher effect size on depression ( HAMD-17 Cohen’s d = 1.33). In our study effect size on self-rated PTSD symptoms was moderate (PCL-5 Cohen’s d = 1.4). The latter is higher than the effect size reported in Philip et al. [5], which was a controlled blinded study reducing placebo effect, and lower than that reported in Nursey et al. [6], which used a clinician administered rating scale limiting the validity of the comparison.

Limitation of our study include: small sample size, only males, and being uncontrolled. Our iTBS paradigm, while it is designed using the same physiological principles, differed from the published iTBS paradigm.

Taken together, this study points out to the need for a larger, controlled studies for TRD co-morbid with PTSD. Furthermore, comparing paradigms that has promise in this population including right sided versus bilateral sequential, utilizing different combination of iTBS and cTBS, would inform the field about the most efficient method to treat TRD in PTSD.

## Data Availability

Data set used in this study is available from corresponding author upon proper request.

## Declarations

LP reports personal fees from Janssen Canada, Otsuka Canada, SPMM Course Limited, UK, Canadian Psychiatric Association; book royalties from Oxford University Press; investigator-initiated educational grants from Janssen Canada, Sunovion and Otsuka Canada outside the submitted work. LP received travel support as a speaker for a meeting at Oxford, UK organized by Magstim limited in 2014. None related to this work.

AMB reports personal fees from Janssen Canada, Avanir and Eisai pharmaceuticals for serving on advisory boards and from Atheneum Partners for providing expert consultation on market assessment. None are related to this work.
